# The internal and external consistency of a speech reception threshold test for isiZulu speakers with normal hearing sensitivity

**DOI:** 10.4102/sajcd.v65i1.556

**Published:** 2018-06-25

**Authors:** Seema Panday, Harsha Kathard, Mershen Pillay, Wayne Wilson

**Affiliations:** 1 Discipline of Audiology, School of Health Sciences, University of KwaZulu-Natal, South Africa; 2 Department of Health and Rehabilitation Sciences, University of Cape Town, South Africa; 3 School of Health and Rehabilitation Sciences, The University of Queensland, Australia

## Abstract

**Background and objectives:**

This study investigated reliability, particularly the internal and external consistency, of a new isiZulu speech reception threshold (SRT) test.

**Methods:**

To examine internal consistency, 21 adult isiZulu speakers with normal hearing sensitivity completed the SRT test using the first and second halves of the SRT wordlist in the same test session. To examine external consistency, a separate 23 adult isiZulu speakers with normal hearing sensitivity completed the SRT test, using the whole word list on two occasions 4 weeks apart. Consistency of SRT test scores in these test conditions was measured using intraclass correlation coefficient analyses (a measure of the consistency or reproducibility of different observations of the same quantity) and Bland and Altman analyses of agreement (a comparison of measurement error with the expected variation amongst subjects).

**Results:**

Intraclass correlation coefficient values ranged from 0.69 to 0.79, showing the isiZulu test scores were highly consistent between the test and retest conditions used in this study. Bland and Altman analyses showed that isiZulu speakers with normal hearing sensitivity can be expected to return isiZulu SRT test scores that differ by no more than 7.5 dB HL – 8.7 dB HL between original and repeat assessments.

**Conclusion:**

The isiZulu SRT test was reliable, showing high internal and external consistency, when used to assess first-language speakers of isiZulu with normal hearing sensitivity. These findings warrant continued development of the isiZulu SRT test for eventual clinical use. This development should include validating this test on first-language speakers of isiZulu with and without hearing loss.

## Introduction

Since its introduction in the 1950s, speech reception threshold (SRT) testing has maintained its place in the basic audiological test battery (Ramkissoon, Proctor, Lansing, & Bilger, 2002). SRT tests typically consist of a list of words presented to a listener who must repeat each word as heard. The level of the words is then altered until the listener correctly repeats 50% of a group of words. This level is considered to be the listener’s SRT, which is used to quantify the listener’s hearing level for speech, cross-check the listener’s pure-tone average threshold from pure-tone audiometry testing and provide diagnostic and prognostic value for medical, surgical and/or rehabilitative management of hearing loss (Gelfand, 2001).

Despite its widely reported clinical value, SRT testing still faces several significant threats to its reliability and validity as a measure of speech reception. Harris et al. (2007) summarised these threats to include the number, type and homogeneity of the words in the test; the accent or dialect of the speaker; and the method and level of stimulus presentation. Panday, Kathard, Pillay and Govender (2007, 2009) provided a description of how these threats may be addressed when developing SRT tests.

Another important threat to the reliability and validity of SRT testing is the listener’s familiarity with the test word stimuli (Lyregaard, 1997; Nissen, Harris, Jennings, Eggett, & Buck, 2005). Such familiarity in SRT testing is best achieved by using words from the first language (or mother tongue) of the target population (Nissen et al., 2005). In this regard, Craig (1997) warned the use of unfamiliar test words on groups of first and second language speakers of a language will result in the second language speakers being less able or unable to hear subtle sound segments and prosodic nuances of the word stimuli. Similarly, Takayanagi, Dirks and Moshfegh (2002) found second language English speakers both with and without hearing impairment required higher intensity for equal recognition of English test words. Such findings suggest that words having a higher frequency of occurrence in the target population, that is, familiar words, are more easily recognised by persons in that population than are words with lower frequencies of occurrence. It also suggests that listeners rely on their higher order cognitive resources such as prosodic, semantic, lexical knowledge of the language and context in order for them to recognise and understand test word stimuli (Lyregaard, 1997; Medwedsky, 2002).

The majority of SRT tests used in audiology have been developed in English using American English spondee words, where a spondee word is a bisyllabic word pronounced with equal stress on each syllable (Ballachanda, 2001; Ramkissoon et al., 2002). The use of these SRT tests on non-English (and even non-American English) speaking populations can be inappropriate both culturally and linguistically (Ramkissoon et al., 2002). Such inappropriate application can adversely affect the interpretation of SRT test results, with non-American English speakers performing poorly on these tests not because they have a true speech reception deficit, but because they are simply unfamiliar (or at least less familiar) with American English (Aleksandrovsky, McCullough, & Wilson, 1998; Harris, Kim, & Egget, 2003; Harris, Nielson, McPherson, Skarzynski, & Egget, 2004; Martin & Hart, 1978; Ramkissoon et al., 2002).

The potential risks of using culturally and linguistically inappropriate SRT tests are particularly relevant in South Africa with its diverse multicultural and multilingual population that has experienced major, recent socio-political change. Such risks have seen calls to develop contextually relevant tests in South Africa not only for items such as SRT tests in audiology, but also for resources across the full spectrum of health and rehabilitation sciences. One such call for audiology and speech pathology was from Pascoe (2011) who defined contextually relevant resources as:

… any tools (assessments, intervention programmes, guidelines and norms) that are available for speech-language therapists and audiologists to use with a specific population in a specific setting, and that have been developed with that population and setting in mind. (pp. 2–5)

Importantly, Pascoe also acknowledged efforts in South Africa to not only develop test materials in indigenous local languages, but also to encourage collaborations amongst researchers, clinicians and the local populations to develop knowledge of the process of these developments.

Attempts to address the need for culturally and linguistically appropriate SRT and other speech audiometry tests in South Africa have generally focused on two areas: the potential for using non-South African language speech audiometry tests and developing new South African language speech audiometry tests. On the potential for using non-South African language speech audiometry tests on South African populations, research has suggested that first-language speakers of South African English with normal hearing thresholds perform well on non-South African English speech recognition tests at suprathreshold levels but perform poorly at threshold levels. This was seen in studies using the National Acoustic Laboratories Arthur Boothroyd (NAL-AB) wordlists in Australian English (Wilson, Jones, & Fridjhon, 1998) and the Central Institute of the Deaf Wordlist 22 (CID W22) in American English (Wilson & Moodley, 2000). While not addressing SRT testing directly, these studies argue against using or adapting non-South African language speech audiometry tests for South African populations.

On the development of new South African language speech audiometry tests, recent research has shown significant promise. This includes the ongoing development of the South African Spondaic (SAS) wordlists in South African English (Hanekom, Soer, & Pottas, 2015) and a non-standardised Tswana wordlist (Khoza, Ramma, Mophosho, & Moroka, 2008). Both of these tests are intended for SRT testing with South African populations with normal hearing thresholds performing better or equivalently on these tests compared to equivalent tests in non-South African languages. It also includes the ongoing development of a speech-in-noise test in Afrikaans (Theunissen, Swanepoel, & Hanekom, 2009). The Hanekom et al. (2015) and Khoza et al. (2008) studies in particular support the need for ongoing efforts to develop culturally and linguistically appropriate SRT tests for South African populations.

While most South African people are multilingual, the most spoken language is isiZulu, with approximately 23% of South Africa’s 53 million people reporting isiZulu as the primary language they speak at home (Census, 2011). IsiZulu is a Nguni language. At least two of its differences to the Germanic language of English are of particular relevance to SRT testing. First, IsiZulu is a tonal language (Rycroft & Ncgobo, 1979) where variations in pitch influence word meaning (other examples of tonal languages include Mandarin and Cantonese). Second, isiZulu does not have spondee words, the word type most commonly used for SRT testing in English. Instead, isiZulu favours trochee words where prominence is placed on the second syllable of a bisyllabic word. This prominence is often achieved by shortening the duration of the second syllable (Cope, 1982), for example, the low tone verb *hamba* (meaning ‘to go’) would typically be pronounced with a shortened ‘ba’. The authors refer the readers to Van der Merwe and Le Roux (2014) for a review of isiZulu lexical tone and syllable structure.

To address the need of culturally and linguistically appropriate SRT tests for South African speakers of isiZulu, Panday et al. (2007, 2009) developed an isiZulu SRT test and Panday, Kathard, Pillay and Wilson (2018) have begun to systematically validate this isiZulu SRT test that considers the linguistic structure of isiZulu. The current version of this isiZulu SRT test consists of a CD recording of 28 common, bisyllabic low-tone isiZulu verb imperatives (spoken by a male first-language speaker of isiZulu).

To date, the development of the CD recording of the isiZulu SRT test has concentrated on its content validity, that is, how well the test represents the content domain it is being designed to measure. To begin, two isiZulu-speaking language interpreters and two tertiary level educators identified 131 commonly used isiZulu words for possible use in an isiZulu SRT test, with 124 of these words subsequently identified as being bisyllabic verbs. Five linguists (each holding a Master’s degree in the linguistics of African Languages) then rated 58 of these bisyllabic verbs as being sufficiently familiar, phonetically dissimilar and low in tone to be potentially suitable for use in the development of an SRT test in isiZulu (Panday et al., 2007). Recorded versions of these 58 bisyllabic words (spoken by an adult male, first-language speaker of isiZulu) were then tested for homogeneity of audibility by playing the words at six intensity levels to 30 isiZulu first-language-speaking adults (aged 18–25 years) with normal hearing (Panday et al., 2009). Homogeneity of audibility was determined by examining the psychometric functions (also known as the performance intensity functions or curves) of each of the 58 word recordings. These functions illustrate how well a speech sample or test item is correctly identified as a function of intensity level. Twenty-eight of the recorded words met the criterion of having a mean slope at 50% intelligibility within 1 SD of the group mean of 5.98%/dB. Finally, an analysis of the prosodic features and pitch contours of these 28 word recordings showed these word recordings conformed to the prosodic pattern apparent within the linguistic structure of isiZulu (Panday et al., 2009).

The present study continues the development and validation of Panday et al.’s (2007, 2009) isiZulu SRT test by determining its reliability when applied to first-language isiZulu speakers with normal hearing.

## Methods

### Research design

This study proceeded in two parts. Part 1 was designed to assess the internal consistency and part 2 was designed to assess the external consistency of the new isiZulu SRT test. Both parts of this study used an observational, repeated measures design.

### Participants

Part 1 of this study conveniently sampled 21 persons (5 males and 16 females; aged 17–51 years, median = 35 years, first quartile = 24 years and third quartile = 44 years), and part 2 conveniently sampled 23 persons (4 males and 19 females; aged 21–58 years, median = 36 years, first quartile = 24.5 years and third quartile = 43 years), from the adult, first-language isiZulu-speaking population of KwaZulu-Natal, South Africa.

All participants in this study were first-language speakers of isiZulu (self-reported) and permanent residents of KwaZulu-Natal, South Africa (as shown on their South African identity documents). They had responded to advertisements in posters and flyers distributed by the researchers in their communities. After three rounds of advertisement, participants were also recruited through family members of enrolled participants and the University of KwaZulu-Natal community. All participants had unremarkable medical and hearing histories (self-reported), hearing thresholds ≤ 25 dB HL at octave frequencies from 250 Hz to 8000 Hz, and normal middle ear pressure and compliance (ASHA, 1988; Roup, Wiley, Safady, & Stoppenbach 1998).

### The isiZulu speech reception threshold wordlist

The ongoing development of the isiZulu SRT wordlist (see Appendix 1) used in this study has been reported by Panday (2006), Panday et al. (2007, 2009) and Panday et al. (2018). Its current recording (Panday et al., 2018) consists of 28 bisyllabic isiZulu low tone verbs shown to satisfy criteria of linguistic familiarity and homogeneity of audibility for isiZulu-speaking adults. These verbs are (in order as per the current recording): *banga, gxeka, cinga, faka, thela, linda, khaba, kheta, thatha, donsa, washa, chela, xola, yonga, yona, veza, wina, khanya, shada, geza, khipa, thola, jeza, qonda, thenga, loya, minya* and *yeka*. Each recorded isiZulu word had been adjusted in level (Panday et al., 2018) to ensure its 50% correct perception score occurs at the mean pure-tone average (2.8 dB HL) of the 30 normally hearing isiZulu first-language-speaking adults (mean age 21.5 years) who participated in Panday et al. (2009).

#### Procedure

Prior to completing the SRT assessments, all participants filled out a case history questionnaire and underwent pure-tone audiometry assessment at octave frequencies from 250 Hz to 8000 Hz using a Grayson-Stradler GSI 61 twin-channel clinical audiometer with TDH-49 telephonic earphones and MX41-AR cushions, and a tympanometric assessment using a GSI Tympstar clinical middle ear analyser. This testing was completed in an isolated Industrial Acoustics Company twin audiometric soundproof booth of double wall construction meeting ANSI (1977) standards.

All SRT testing was conducted using the isiZulu SRT test CD, a Technics (SLPG390) CD player and the audiometer and booth described above. This testing was conducted by an isiZulu first-language-speaking audiologist with 6 years of clinical experience who had been trained by the present study’s first author (S.P.). Each SRT test began with the audiologist showing the participant a printed copy of the words (in a randomised order) contained in the isiZulu SRT test as it was being presented on that occasion. The audiologist and the participant then read aloud each word with the participant being given the opportunity to clarify any words with which he or she was unfamiliar (as recommended by ASHA, 1988). The audiologist then placed headphones on the participant and played the isiZulu SRT test instructions from the CD at 30 dB SL.

To measure each participant’s SRT, the audiologist followed a modified version of the Chaiklin and Ventry (1964) descending method cited in Gelfand (2001) for SRT testing. This method was modified by changing the starting level from 25 dB SL relative to a two-frequency pure-tone average to 10 dB SL relative to the three-frequency (0.5 kHz, 1 kHz and 2 kHz) pure-tone average. This was performed to reduce test time in expectation of the participants having normal pure-tone thresholds.

To complete part one of this study investigating the internal consistency of the isiZulu SRT test, the isiZulu SRT word list was split such that the first 14 words formed Test A (banga, cinga, thela, khaba, thatha, washa, xola, yona, khanya, geza, thola, qonda, thenga and minya) and the last 14 words formed Test B (gxeka, faka, linda, kheta, donsa, chela, yonga, veza, wina, shada, khipa, jeza, loya and yeka). The SRT for each participant was then determined for Test A and Test B. These SRT measures were conducted sequentially within each participant and for right and left ear stimulation separately. The order of testing (Test A then Test B or vice versa) and ears tested (right then left or vice versa) was counterbalanced amongst participants.

To complete part two of this study investigating the external consistency of the isiZulu SRT test, the SRT for each participant was determined using all 28 words of the isiZulu SRT test on two occasions, 4 weeks apart. These SRT values were determined for right ear stimulation only.

### Data analysis

Descriptive statistics were calculated for each participant’s SRT score for each variation of the SRT assessment. These scores were confirmed as meeting parametric assumptions by inspecting their histograms, box-and-whisker plots and Q-Q plots.

For both parts one and two of this study, the consistency of the SRT scores obtained, were assessed using two methods. The first method was intraclass correlation coefficients (ICCs) using a two-way random model (2, 1) and the 95% confidence intervals (CI) for these ICCs. The second method was the Bland and Altman method for assessing agreement. This includes calculations of the mean difference between measures ($\bar{d}$), the 95% CI for $\bar{d}$, the standard deviation of the differences (SD_diff_), the 95% limits of agreement and a reliability coefficient.

The measures of intraclass correlation were completed according to the Shrout and Fleiss (1979), Rankin and Stokes (1998) and Bartlett and Frost (2008). The strength of reliability indicated by the ICC values was determined using the general (although arbitrary) guidelines reported by Landis and Koch (1977) of values <0 indicating poor agreement, 0.01–0.20 indicating slight agreement, 0.21–0.40 indicating fair agreement, 0.41–0.60 indicating moderate agreement, 0.61–0.80 indicating substantial agreement and 0.81–1.00 indicating almost perfect agreement.

The Bland and Altman method of assessing agreement was conducted according to Altman (1991), Bland (1987) and Bland and Altman (1986). The strength of reliability based on this assessment was determined by considering the argument that the reliability of a measure should reflect the true variability of that measure in the target population (Riddle, Finucane, Rothstein, & Walker, 1989; Streiner & Norman, 1995). This argument suggests that reliability is relative and should reflect how well a measurement can differentiate individuals in the target population. In this regard, reliability (or measurement error) should be contrasted with the expected variation amongst the subjects being tested (Streiner & Norman, 1995). A brief, narrative review of the literature on SRT testing, using words, suggested that a variability of ± 5 dB would be a clinically acceptable variation in measured SRT values within subjects (ANSI, 2004; ASHA, 1988; Caswell, 2013; Hallgren, Larsby, & Arlinger, 2006; Neuman, Baumann, Sick, Euler, & Weigerber, 2012). As a result, any differences of ± 5 dB in the mean difference ($\bar{d}$) scores in any of the SRT assessments conducted in the present study (in the split-half or test-retest analyses) were considered to indicate that the SRT score was reliable as the difference ($\bar{d}$) score was no greater than the expected variation within subjects.

## Ethical consideration

Unconditional ethical clearance was granted by the Faculty of Health Sciences Human Research Ethics Committee of the University of Cape Town to conduct the study (clearance number: HREC 652/2012).

## Results

**FIGURE 1:**
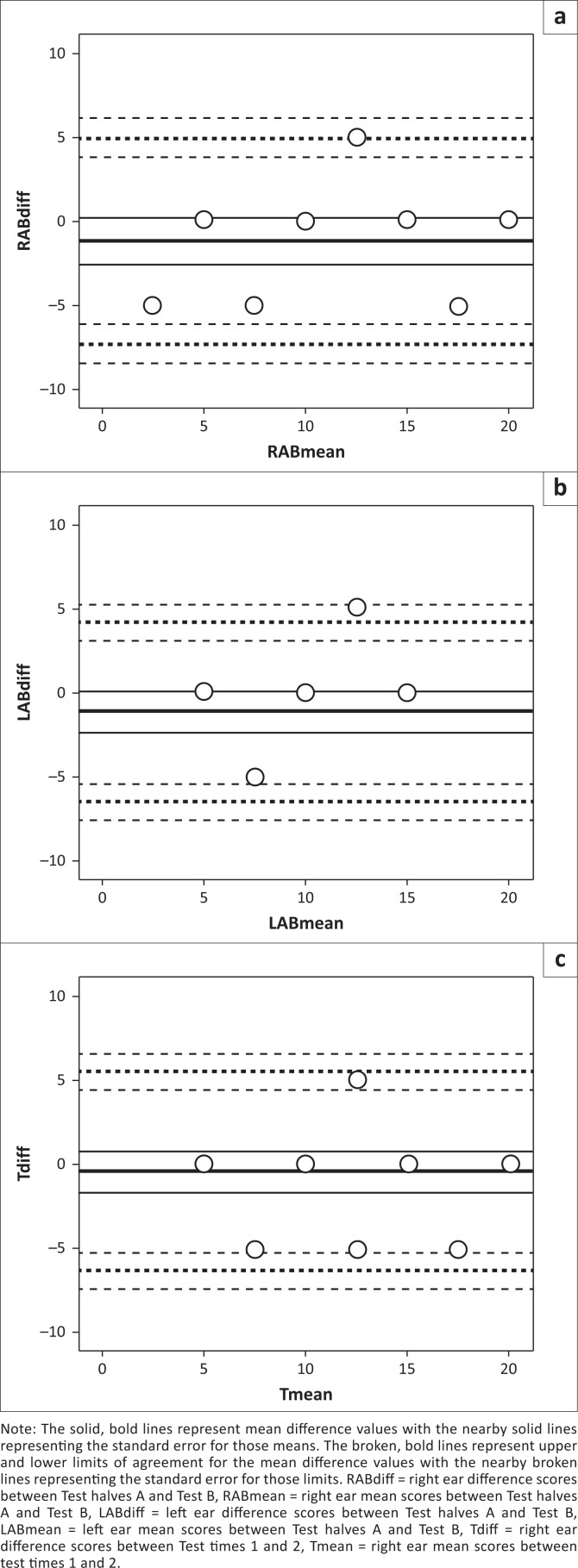
Bland and Altman plots to examine the consistency of the isiZulu SRT test results. Internal consistency was considered by comparing scores between Test halves A and Test B for the right ear ([plot] a) and left ear ([plot] b). External consistency was considered by comparing whole test scores between Test times 1 and 2 for the right ear ([plot] c).

Table 1 shows the SRT measurements and Table 2 shows the ICCs (2, 1) and Bland and Altman test results for parts one and two of the study. Figure 1 shows the Bland and Altman plots for parts one and two of the study. All ICC (2, 1) values were in the range of 0.69–0.79 indicating substantial agreement (Landis & Koch, 1977). The Bland and Altman test results showed no evidence of systematic variability in any SRT difference scores. Slight negative trends in the difference values were noted although these trends were not considered to be significant relative to the magnitude of the measurement. The observed difference scores ranging from +5 dB HL to -5 dB HL would not be considered as important in clinical measurement of SRT (ANSI 2004; ASHA, 1988; Caswell, 2013; Hallgren et al., 2006; Neuman et al., 2012). The coefficient of repeatability ranged from 7.5 to 8.7 for the SRT measures in both parts of the study. These coefficients indicate that the differences between two measurements of an SRT value obtained using any one of the three variations of SRT measurement used in the present study can be expected to differ by no more than 7.5 dB HL – 8.7 dB HL on 95% of occasions.

**TABLE 1:** Speech reception threshold measurements for parts one (internal consistency) and two (external consistency) of the study.

Variable	Part 1 (split-half)				Part 2 (test-retest)	
	Test A (1st half of list)		Test B (2nd half of list)		Test (whole list)	Retest (whole list)
	RE	LE	RE	LE	RE	RE
Mean	9.1	9.5	10.2	10.7	10.7	11.1
SD	5.2	4.2	4.0	2.9	4.6	4.51
Minimum	0.0	5.0	5.0	5.0	5.0	50
Maximum	20.0	15.0	20.0	15.0	20.0	200

SD, standard deviation; RE, right ear; LE, left ear.

**TABLE 2:** Intraclass correlation coefficients (2,1) and Bland and Altman tests for parts 1 (internal consistency) and 2 (external consistency) of the study.

Variable	ICC (2,1)		Bland and Altman					Reliability coefficient
	ICC coefficient	95% CI	$\bar{d}$ (dB HL)	SE of $\bar{d}$	95% CI for $\bar{d}$	SD_diff_	95% LOA	
Part 1 (split-half) – RE	0.76	0.49 to 0.89	−1.19	0.68	−2.61 to 0.23	3.12	−7.31 to 4.93	8.7
Part 1 (split-half) – LE	0.69	0.37 to 0.86	−1.19	0.59	−2.42 to 0.04	2.69	−6.47 to 4.09	7.5
Part 2: (test-retest) – RE	0.79	0.57 to 0.91	−0.43	0.62	−1.72 to 0.86	2.98	−6.28 to 5.41	8.3

Note: $\bar{d}$ is the mean difference; SE of $\bar{d}$ is the standard error of the mean difference; 95% CI for d is the 95% confidence interval for the mean difference.

SD_diff_, standard deviation of the differences; LOA, limits of agreement; ICC, intraclass correlation coefficients.

## Discussion

The isiZulu SRT test was reliable, showing high internal and external consistency, when used to assess first-language speakers of isiZulu with normal hearing sensitivity.

The high internal consistency was shown by the split-half analysis results showing substantial absolute agreement on ICC (2, 1) analyses, and the range of difference ($\bar{d}$) scores being within the ±5 dB variation in SRT scores expected within subjects in a clinical setting on the Bland Altman analyses, for both right and left ear stimulation. These results show that both the first and second halves of the isiZulu SRT test wordlist contributed equally to the measurement of SRT in the present study’s isiZulu-speaking subjects with normal hearing sensitivity. While not recommended, these results also suggests that the isiZulu SRT test could be used in half-list form (i.e. only half of the words in the list to be used during an assessment) should such a need arise in a clinical setting.

The high external consistency was shown by test-retest analysis results showing substantial agreement on ICC (2, 1) analysis, and the range of difference ($\bar{d}$) scores on the Bland Altman analyses being within the ±5 dB variation in SRT scores expected within subjects in a clinical setting, for the right ear stimulation used in this part of the study. These results show that test scores on the isiZulu SRT test can be expected to remain stable in isiZulu-speaking subjects with normal hearing sensitivity who experience no changes in their hearing over a 4-week period. Such a finding should prove useful in clinical settings where serial monitoring of SRTs is needed up to a monthly basis.

At least four limitations are noted in the present study that should be considered before generalising the results to the wider isiZulu-speaking population. First, the two parts of the study sampled 21 and 23 participants, respectively, with both samples being non-random in nature. Second, the method of obtaining the SRT scores was modified from that described by Chaiklin and Ventry (1964) cited in Gelfand (2001), which may not immediately generalise to SRT scores obtained using other methods. Third, the split-half analysis of the isiZulu SRT test was conducted by splitting its wordlist into half by word order (a simple split of the first 12 and the last 12 words in the whole SRT wordlist). Other methods such as splitting the words by odd and even positions could have obtained different results. Finally, the Bland and Altman results were deemed to support the consistency of the SRT test results on the basis that variability of ±5 dB in SRT score would be clinically acceptable. A requirement for less variability would change the interpretation of these Bland and Altman test results.

The high reliability, both for internal and external consistency, of the new isiZulu SRT test showed in first-language speakers of isiZulu with normal hearing sensitivity warrants the continued development of this test for eventual clinical use. The 28 isiZulu wordlist recording is not yet available for clinical use as further research is needed to validate its use on first-language speakers of isiZulu with and without hearing loss. As many of the previous studies conducted locally in South Africa focused on the development aspect of speech audiometry tests, the future validity testing of this isiZulu test will contribute to local literature both in terms of methods followed and clinical application of the test for hearing and hearing impaired individuals.

## Conclusion

The isiZulu SRT test was reliable, showing high internal and external consistency, when used to assess first-language speakers of isiZulu with normal hearing sensitivity.

## Appendix 1

The 28 bisyllabic isiZulu low tone verbs in the isiZulu SRT wordlist used in the present study:

1. banga
2. gxeka
3. cinga
4. faka
5. thela
6. linda
7. khaba
8. kheta
9. thatha
10. donsa
11. washa
12. chela
13. xola
14. yonga
15. yona
16. veza
17. wina
18. khanya
19. shada
20. geza
21. khipa
22. thola
23. jeza
24. qonda
25. thenga
26. loya
27. minya
28. yeka
